# The Preferable Climate for Phthisis

**Published:** 1888-12

**Authors:** 


### THE PREFERABLE CLIMATE FOR PHTHISIS.

In this paper, read before the late International Medical Congress, Dr. Charles Denison attempts to show the attributes which in his experience go to make up the ideal climate for consumptives. The essential features of this climate he considers to be: 1, dryness, as opposed to moisture; 2, coolness or cold, as preferable to warmth or heat; 3, rarefaction, as opposed to sea-level pressure; 4, sunshine, as opposed to cloudiness; 5, variability of temperature, as opposed to equability.

Dr. Denison has given great attention to the subject of climate in this disease, hence his papers are worthy of careful attention.

